# Impact of an Educational Leaflet About Asymptomatic Bacteriuria and Urinary Tract Infection on Antibiotic Preferences Among US Adults ≥65 Years: An Online Randomized Controlled Survey Experiment

**DOI:** 10.1093/ofid/ofaf690

**Published:** 2025-12-15

**Authors:** Alistair Thorpe, Rachael A Lee, Julia E Szymczak, Madeline C Farrell, Karen Howard, Brandi M Muller, Andrea T White, Angela Fagerlin, Valerie M Vaughn

**Affiliations:** Department of Population Health Sciences, Spencer Fox Eccles School of Medicine at University of Utah, Salt Lake City, Utah, USA; Division of Infectious Diseases, Department of Medicine, UAB School of Medicine, Birmingham, Alabama, USA; Division of Infectious Diseases, Department of Medicine, Birmingham VA Medical Center, Birmingham, Alabama, USA; Division of Epidemiology, Department of Internal Medicine, Spencer Fox Eccles School of Medicine at University of Utah, Salt Lake City, Utah, USA; Department of Pharmacy, University of Utah Health, Salt Lake City, Utah, USA; Division of Epidemiology, Department of Internal Medicine, Spencer Fox Eccles School of Medicine at University of Utah, Salt Lake City, Utah, USA; Department of Internal Medicine, Spencer Fox Eccles School of Medicine at University of Utah, Salt Lake City, Utah, USA; Department of Population Health Sciences, Spencer Fox Eccles School of Medicine at University of Utah, Salt Lake City, Utah, USA; Salt Lake City VA Informatics Decision-Enhancement and Analytic Sciences (IDEAS) Center for Innovation, Salt Lake City, Utah, USA; Department of Internal Medicine, Spencer Fox Eccles School of Medicine at University of Utah, Salt Lake City, Utah, USA

**Keywords:** antibiotic stewardship, antimicrobial resistance, asymptomatic bacteriuria, patient education, urinary tract infection

## Abstract

**Background:**

Adults aged ≥65 years are at high risk of harm from antibiotic misuse due to misdiagnosis of asymptomatic bacteriuria (ASB) as urinary tract infection (UTI). Alongside strategies to improve prescribing, patients should be informed and empowered to discuss the harms/benefits of antibiotic treatment. We tested whether a patient-focused educational leaflet improved reported willingness to avoid antibiotics when not clinically necessary.

**Methods:**

In an online randomized controlled survey experiment, US adult respondents aged ≥65 years read a scenario of themselves as an asymptomatic patient with a positive urine test before a nonurologic surgical procedure. They were assigned to 1 of 4 conditions, which varied by educational leaflet provision and surgeons' treatment recommendation for antibiotics. The primary outcome was respondents' comfort with not taking antibiotics for ASB. Secondary outcomes were reported misperceptions of ASB as UTI and knowledge.

**Results:**

Of the 504 respondents (completion = 89%), the mean age was 72, 64.5% identified as male, 53.4% identified as Non-Hispanic White, and 35.7% reported prior antibiotic prescriptions for UTI. In response to the vignette, respondents shown the leaflet were more comfortable not taking antibiotics (*P* < .001), were less likely to misperceive ASB as a UTI (*P* < .001), and displayed greater knowledge (*P* < .001). Respondents told that the surgeon recommends antibiotics were less comfortable not taking antibiotics (*P* = .013) and more likely to misperceive ASB as UTI (*P* < .001).

**Conclusions:**

In an online randomized controlled survey experiment, a patient-centered educational leaflet decreased reported desires to take antibiotics for ASB and improved knowledge among US adults age ≥65 years. Patient-focused education may prepare patients to engage in antibiotic treatment decisions.

Unnecessary antibiotic use harms patients, burdens health care systems, and promotes antibiotic resistance [1]. Asymptomatic bacteriuria (ASB) describes the presence of bacteria in the urine of a patient without signs of infection. ASB is often misdiagnosed as urinary tract infection (UTI), leading to unnecessary antibiotic use that does not improve clinical outcomes for most patients [2–7]. Given the patient harms and health care costs associated with unnecessary antibiotic use [2–7], guidelines recommend against testing for, or treating, ASB, with few exceptions (eg, certain urologic conditions) [5]. Despite these recommendations, high levels of misuse persist, particularly in hospitalized patients, where up to 80% of ASB is treated [2, 8].

Older adults (≥65 years) are more likely to have ASB and to be unnecessarily treated [2, 9–14]. Because ASB is common with advancing age, urine tests for older adults are often “positive or abnormal,” which motivates clinicians, patients, and caregivers to initiate antibiotics [15]. This initial misuse of antibiotics triggers recurring (eg, “this is how I felt last time I had a UTI”) and escalating cycles of antibiotic prescribing (eg, to treat increasingly resistant ASB), putting patients at greater risk of adverse outcomes [16].

Educating patients and their caregivers about ASB and empowering them to discuss antibiotic treatment risks/benefits represents a key goal of patient-centered care and has significant potential to help reduce unnecessary antibiotic use [17]. Most efforts to improve antibiotic prescribing for ASB have focused on improving clinician decision-making [18, 19]. As a result, comparatively less focus has been given to the patient and caregiver perspective [17], despite their established influence on clinicians' prescribing behaviors [20].

One often cited reason why clinicians prescribe antibiotics when they are not needed is to prevent potential patient and caregiver dissatisfaction and avoid conflict with those who may expect antibiotics [21–24]. Prior research demonstrates that when a clinician perceives a patient as expecting an antibiotic they are more likely to prescribe antibiotics, even when the patient or caregiver does not actually want them [25]. Notably, clinicians who are more confident in their ability to reassure patients and caregivers when antibiotics are not needed are less likely to overprescribe, especially when supported with effective patient education resources [26–28]. Educational resources for reassuring patients and caregivers that antibiotics are not needed for ASB may therefore help clinicians avoid unnecessary antibiotic prescribing while also improving patient outcomes and care experiences.

However, few evidence-based, patient-facing educational resources exist for older patients about ASB-related care [29]. Previously, we worked with patients, caregivers, and clinicians via iterative user-centered design to develop an educational leaflet on UTI, ASB, and the benefits/risks of antibiotic treatment for patients aged ≥65 years and their caregivers [30]. In the present study, we conducted an online survey experiment to test the impact of the educational leaflet on respondents' reported willingness to avoid unnecessary antibiotics as well as their knowledge about ASB, UTI, and antibiotics. As patients and caregivers report receiving inconsistent guidance from clinicians [17], we also examined the impact of a surgeon's recommendation to take antibiotics alongside the educational leaflet. We expected that respondents who received the educational leaflet about ASB would be more comfortable not taking antibiotics for ASB (H1), whereas those who were told that the surgeon recommended antibiotics would be less comfortable (H2).

## METHODS

### Study Population and Recruitment

From March 21 through April 23, 2024, respondents were recruited to take part in an online survey using Qualtrics Online Panels (Provo, UT, USA; qualtrics.com). We planned to recruit 500 respondents via online invitation, which would provide sufficient statistical power (90%) to detect small-to-medium effect sizes (*f* = .17), with significance set at .05 [31]. Potential respondents were recruited by Dynata (Shelton, CT, USA; dynata.com), a commercial market research company with diverse pools of individuals who have agreed to be invited to take part in online survey studies.

The study was administered in English and received approval (deemed exempt) from the Institutional Review Board at the University of Utah (IRB_00167676). Respondents consented to participate and were compensated for their participation based on the terms of their agreement with Dynata.

#### Inclusion

Potential respondents from Dynata's invitation pool were eligible if they were US-residing, English-speaking adults aged ≥65 years with internet access. We employed nonprobability sampling using quotas for self-reported age, gender identity, racial/ethnic identity, and US Census region to ensure adequate representation within our sample (Supplementary Data).

#### Exclusion

For this study, respondents aged <65 years were not included. To enhance data quality, we also excluded respondents post hoc who finished in <5 minutes and those whose open-text answers raised doubts about the validity of their responses.

### Procedure

**Figure 1.**
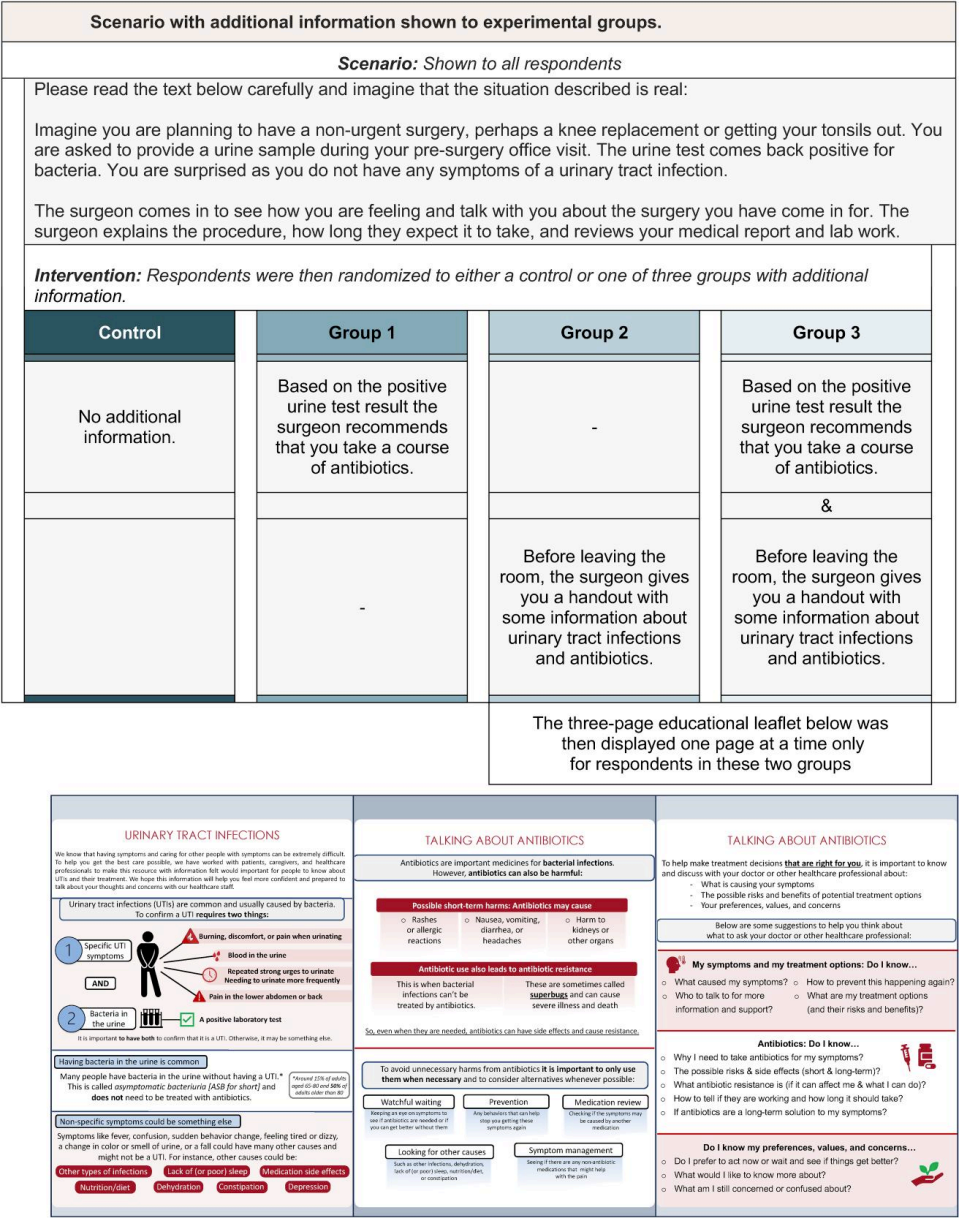
Scenario with additional information shown to experimental groups.

Respondents were shown a scenario describing themselves as an asymptomatic patient with a positive urine test during prescreening for a nonurgent, nonurologic surgery (Figure 1; Supplementary Data). The scenario was designed by the study team to represent a situation in which, according to clinical guidelines, such a patient has ASB, not a UTI, and should not receive antibiotics [5]. Respondents did not have the opportunity to go back at any point in the survey to review the information they had seen or change their initial responses. This study follows the EQUATOR Network Guideline for RepOrting Vignette Experiments (GROVE) reporting guideline [32], with details available in the Supplementary Data.

In a 2 × 2 between-group experimental design, we examined the effects of a 3-page PDF educational leaflet about UTIs, ASB, and antibiotics (not provided vs provided) alongside a surgeon's treatment recommendation (none given vs recommended to take antibiotics). The educational leaflet had been previously developed by members of the study team with input from patients, caregivers, and clinicians (Figure 1) [30]. Within this factorial design, respondents were randomly allocated to either a control group or 1 of 3 intervention groups, as shown in Table 1.

**Table 1.** Respondent Characteristics and Outcome Measures by Group Assignment

	Control	Group 1	Group 2	Group 3	
	No Additional Information	Surgeon Recommends Antibiotics	Received Educational Leaflet	Surgeon Recommends & Received Leaflet	Total Respondents
Characteristic No. (%)	126 (25.0)	132 (26.2)	118 (23.4)	128 (25.4)	504
Age, y					
Mean (SD)	71.5 (5.3)	72.0 (5.4)	71.2 (4.5)	72.5 (5.0)	71.8 (5.1)
65 to 69	56 (44.4)	52 (39.4)	58 (49.2)	45 (35.2)	211 (41.9)
70 to 74	36 (28.6)	44 (33.3)	30 (25.4)	39 (30.5)	149 (29.6)
75 to 79	21 (16.7)	20 (15.2)	26 (22.0)	30 (23.4)	97 (19.2)
80 to 85	9 (7.1)	12 (9.1)	3 (2.5)	13 (10.2)	37 (7.3)
≥85	4 (3.2)	4 (3.0)	1 (0.8)	1 (0.8)	10 (2.0)
Gender identity, No. (%)					
Male	79 (62.7)	86 (65.2)	77 (65.3)	83 (64.8)	325 (64.5)
Female	47 (37.3)	46 (34.8)	41 (34.7)	45 (35.2)	179 (35.5)
Racial/ethnic identity, No. (%)					
Non-Hispanic Black	26 (20.6)	23 (17.4)	17 (14.4)	22 (17.2)	88 (17.5)
Non-Hispanic White	59 (46.8)	66 (50.0)	72 (61.0)	72 (56.2)	269 (53.4)
Hispanic	25 (19.8)	33 (25.0)	20 (16.9)	26 (20.3)	104 (20.6)
Any other race or ethnicity	16 (12.7)	10 (7.6)	8 (6.8)	8 (6.2)	42 (8.3)
Missing	0 (0.0)	0 (0.0)	1 (0.8)	0 (0.0)	1 (0.2)
Region, No. (%)					
Northeast	20 (15.9)	25 (18.9)	24 (20.3)	21 (16.4)	90 (17.9)
Midwest	32 (25.4)	35 (26.5)	31 (26.3)	38 (29.7)	136 (27.0)
South	39 (31.0)	29 (22.0)	31 (26.3)	35 (27.3)	134 (26.6)
West	35 (27.8)	43 (32.6)	31 (26.3)	34 (26.6)	143 (28.4)
Missing	0 (0.0)	0 (0.0)	1 (0.8)	0 (0.0)	1 (0.2)
Prior ABX prescription for UTI					
No	76 (60.3)	82 (62.1)	67 (56.8)	77 (60.2)	302 (59.9)
Yes	41 (32.5)	47 (35.6)	44 (37.3)	48 (37.5)	180 (35.7)
Unsure	9 (7.1)	3 (2.3)	7 (5.9)	3 (2.3)	22 (4.4)
Outcome measures: scenario in which the patient has ASB, not a UTI, and should not receive antibiotics					
Comfort with not taking antibiotics: “How would you feel about NOT taking antibiotics in this situation?”					
Mean (SD)	2.3 (1.0)	2.0 (0.9)	2.6 (0.9)	2.5 (0.9)	2.3 (0.9)
1 (very uncomfortable)	29 (23.0)	44 (33.3)	15 (12.7)	23 (18.0)	111 (22.0)
2 (uncomfortable)	48 (38.1)	50 (37.9)	37 (31.4)	42 (32.8)	177 (35.1)
**3 (comfortable) [coded as comfortable/very comfortable]**	34 (27.0)	33 (25.0)	48 (40.7)	45 (35.2)	160 (31.7)
**4 (very comfortable) [coded as comfortable/very comfortable]**	15 (11.9)	5 (3.8)	18 (15.3)	18 (14.1)	56 (11.1)
Misperception of ASB as UTI: “Based on this scenario, do you think you have a urinary tract infection?”					
0 (no)	46 (36.5)	36 (27.3)	84 (71.2)	81 (63.3)	247 (49.0)
**1 (yes) [coded as incorrect/misperception]**	31 (24.6)	55 (41.7)	12 (10.2)	26 (20.3)	124 (24.6)
2 (I don’t know)	49 (38.9)	41 (31.1)	22 (18.6)	21 (16.4)	133 (26.4)
Knowledge about ASB, UTI, antibiotics					
Mean (SD)	1.7 (1.2)	1.4 (1.2)	2.8 (1.1)	2.6 (1.3)	2.1 (1.3)
If someone has bacteria in their urine, that means that they have a urinary tract infection.					
**Disagree [coded as correct]**	20 (15.9)	15 (11.4)	55 (46.6)	56 (43.8)	146 (29.0)
Agree	42 (33.3)	46 (34.8)	45 (38.1)	46 (35.9)	179 (35.5)
Not sure	64 (50.8)	71 (53.8)	17 (14.4)	26 (20.3)	178 (35.3)
Missing	0 (0.0)	0 (0.0)	1 (0.8)	0 (0.0)	1 (0.2)
Bacteria in the urine does not always need to be treated with antibiotics.					
Disagree	29 (23.0)	23 (17.4)	15 (12.7)	22 (17.2)	89 (17.7)
**Agree [coded as correct]**	47 (37.3)	44 (33.3)	83 (70.3)	85 (66.4)	259 (51.4)
Not sure	50 (39.7)	65 (49.2)	20 (16.9)	21 (16.4)	156 (31.0)
To confirm a bacterial urinary tract infection, you need to have both specific symptoms and a positive test for bacteria in the urine.					
Disagree	15 (11.9)	25 (18.9)	6 (5.1)	18 (14.1)	64 (12.7)
**Agree [coded as correct]**	68 (54.0)	56 (42.4)	101 (85.6)	96 (75.0)	321 (63.7)
Not sure	43 (34.1)	51 (38.6)	10 (8.5)	14 (10.9)	118 (23.4)
Missing	0 (0.0)	0 (0.0)	1 (0.8)	0 (0.0)	1 (0.2)
Symptoms like fever, confusion, feeling tired or dizzy, a change in color or smell of urine, or a fall could have many other causes and might not be a UTI.					
Disagree	8 (6.3)	14 (10.6)	11 (9.3)	6 (4.7)	39 (7.7)
**Agree [coded as correct]**	77 (61.1)	75 (56.8)	94 (79.7)	102 (79.7)	348 (69.0)
Not sure	41 (32.5)	43 (32.6)	13 (11.0)	20 (15.6)	117 (23.2)

Bold font indicates responses coded as correct.

Abbreviations: ABX, antibiotic; ASB, asymptomatic bacteriuria; UTI, urinary tract infection.

### Measures

The primary outcome was the respondents' self-reported comfort with not taking antibiotics based on the scenario, measured using a single item (“How would you feel about NOT taking antibiotics in this situation?”) on a 4-point scale (1 = “very uncomfortable,” 2 = “uncomfortable,” 3 = “comfortable,” 4 = “very comfortable”). Respondents who selected 1 or 2 were then asked “Could you tell us more about why you would feel that way?” and could enter their answer in an open text box.

Secondary outcomes were the misperception of ASB as UTI by respondents (“Based on this scenario, do you think you have a urinary tract infection?”; 0 = “no,” 1 = “yes,” 3 = “I don’t know”) and their knowledge of UTIs, ASB, and antibiotics (4 items; eg, “If someone has bacteria in their urine, that means that they have a urinary tract infection”; 0 = “disagree,” 1 = “agree,” 2 = “not sure”).

Respondents who were shown the educational leaflet were given 6 additional questions asking their perceptions of the educational leaflet: (1) how useful they found it, (2) how easy it was to understand, (3) the accuracy of the information, (4) its relevance to them, (5) how interesting they found it, and (6) how well designed it was. All survey measures for this study can be found in the Supplementary Data.

### Analyses

For the primary analyses, we used a 2-way factorial analysis of variance to examine respondents' reported comfort with not taking antibiotics in the scenario across experimental groups. The same analyses were used for assessing overall knowledge scores across experimental groups. To compare the proportions of respondents who self-reported that they believed they had a UTI (ie, they misperceived ASB as UTI), we used logistic regression.

To assess whether our findings were robust to demographic characteristics, we repeated the analyses described above while accounting for respondent gender (although ASB is more common in older women, we included both men and women so we could assess whether the leaflet could be relevant across the broader population undergoing preoperative evaluation) and reported history of antibiotic prescription for UTI. All analyses were performed using RStudio statistical software, version 2024.04.0, with statistical significance set at .05 (2-sided).

## RESULTS

### Sample Characteristics

A total of 743 US adults aged ≥65 years accessed the survey. Of those, 168 were redirected out of the survey because their demographic quota within the study had already been filled. Out of the remaining 575 respondents, a total of 511 completed the survey (completion rate = 88.9%), with all taking ≥5 minutes to do so. We excluded 7 respondents who provided indecipherable responses in the open text boxes, resulting in a final analytic sample of 504 respondents.

Respondents in the final sample had a mean age of 72 years, 64.5% identified as male, 53.4% identified as Non-Hispanic White, and 35.7% reported having previously been prescribed antibiotics for a UTI. The full characteristics of the sample are reported in Table 1.

### Primary Outcome

**Figure 2.**
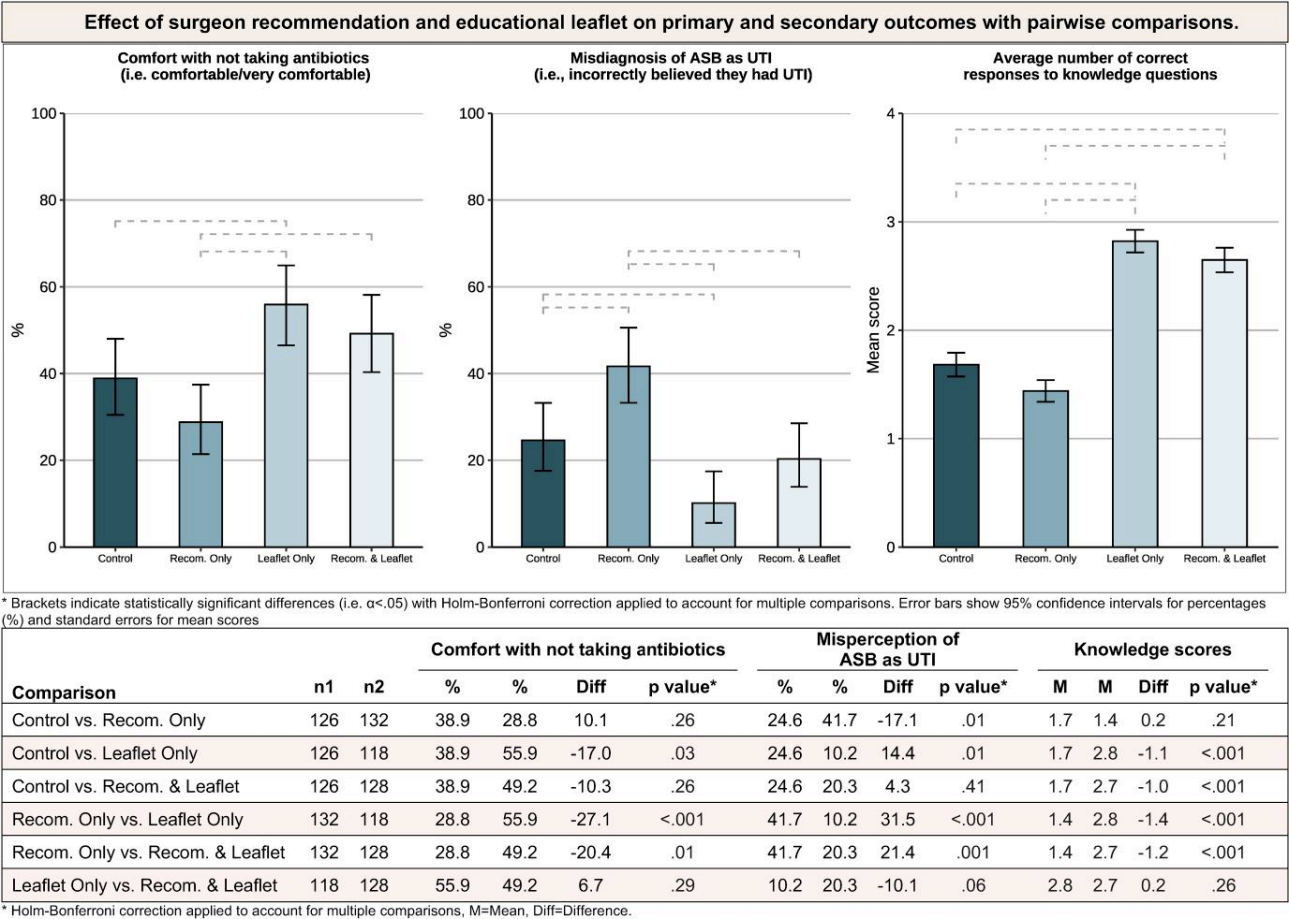
Effect of surgeon recommendation and educational leaflet on primary and secondary outcomes with pairwise comparisons.

As shown in Table 1 and Figure 2, 216 respondents (42.9%) reported that they would be comfortable or very comfortable not taking antibiotics based on the scenario. This differed by group: control group, 38.9% (49); surgeon-recommended antibiotics, 28.8% (38); educational leaflet, 55.9% (66); and 49.2% (63) in the group with both the surgeon recommending antibiotics and the educational leaflet. Open-text responses are available in the Supplementary Data.

In the primary analyses (Table 2), respondents who received the educational leaflet were more comfortable not taking antibiotics (mean [M] ± SD = 2.5 ± 0.9) than those who did not receive the leaflet (M ± SD = 2.1 ± 0.9; *P* < .001). Respondents who were told the surgeon recommends antibiotics were less comfortable not taking antibiotics (M ± SD = 2.2 ± 0.93) than those who did not receive any recommendation (M ± SD = 2.4 ± 0.9; *P* = .013). We found no evidence of an interaction between the educational leaflet and surgeon recommendation (*P* = .35). These findings did not differ when accounting for respondent gender or reporting having previously been prescribed antibiotics for UTI.

**Table 2.** Main Effects and Interactions Across Primary and Secondary Outcomes

	None	Present				Comfort With Not Taking Antibiotics	
	M (SD)	M (SD)	Diff	T Statistic	*P* Value	F Statistic	*P* Value
Effect of surgeon recommendation	2.4 (0.9)	2.2 (0.9)	0.2	2.5	.013	6.4	.011
Effect of leaflet	2.1 (0.9)	2.5 (0.9)	−0.4	−4.7	<.001	22.4	<.001
Interaction	…	…	…	…	…	0.9	.347
	None	Present				Misperception of ASB as UTI	
	No. (%)	No. (%)	Diff, %	χ ^2^	*P* Value	Odds Ratio	*P* Value
Effect of surgeon recommendation	43 (17.6)	81 (31.1)	−13.5	11.7	.001	0.35	.004
Effect of leaflet	86 (33.3)	38 (15.4)	17.9	20.8	.001	2.19	.004
Interaction	…	…	…	…	…	1.03	.951
	None	Present				Knowledge About ASB, UTI, Antibiotics	
	M (SD)	M (SD)	Diff	T Statistic	*P* Value	F Statistic	*P* Value
Effect of surgeon recommendation	2.2 (1.3)	2.0 (1.4)	0.2	1.7	.096	3.4	.065
Effect of leaflet	1.6 (1.2)	2.7 (1.2)	−1.2	−10.9	<.001	119.8	<.001
Interaction	…	…	…	…	…	0.1	.746

### Secondary Outcome

#### Misperceptions of UTI

Overall, 124 (24.6%) respondents misperceived ASB as UTI based on the scenario, a clinically incorrect response given the information provided. Across groups, incorrect UTI beliefs were reported by 31 respondents in the control group (24.6%), 55 in the surgeon recommendation group (41.7%), 12 in the educational leaflet group (10.2%), and 26 in the group with both the surgeon recommendation and the educational leaflet (20.3%). Respondents who received the educational leaflet were less likely to incorrectly report that they had a UTI based on the scenario (15.4%) than those who did not receive the leaflet (33.3%; *P* = .004). Respondents who were told that the surgeon recommends antibiotics, however, were more likely to incorrectly report that they had a UTI (31.1%) than those who did not receive any recommendation from the surgeon (17.6%; *P* = .004). We found no evidence of an interaction between the educational leaflet and surgeon recommendation (*P* = .95).

#### Knowledge

A total of 78 (16%) of respondents did not answer any of the 4 knowledge questions correctly, with the number of correct responses distributed as follows: 1 (18%), 2 (25%), 3 (22%), 4/all (20%). Overall, most respondents were aware that nonspecific symptoms could have many causes other than UTI (348 of 504, 69.0%) and that to confirm a UTI requires both specific symptoms and a positive urine test (321 of 504, 63.8%). Just over half of respondents (259 of 504, 51.4%) were aware that bacteria detected in the urine does not always need to be treated with antibiotics, while less than a third (146 of 504, 29.0%) were aware that bacteria detected in the urine does not by itself indicate a UTI.

There was substantial heterogeneity in the proportion of respondents giving correct responses across groups (Table 2). For instance, the correct response that confirming a UTI requires both specific symptoms and a positive urine test was only given by 56 respondents in the surgeon recommendation group (42.4%) as compared with 101 respondents in the educational leaflet group (86.3%). The knowledge questions demonstrated acceptable reliability (Cronbach's alpha = .7), so they were averaged to create a composite knowledge score to compare across groups. From these analyses, respondents who received the educational leaflet answered more questions correctly (M ± SD = 2.7 ± 1.2) than those who did not (M ± SD = 1.6 ± 1.2; *P* < .001). We found no evidence that the surgeon recommendation affected knowledge (*P*  *=* .07) nor of an interaction between the educational leaflet and surgeon recommendation (*P* = .75).

### Educational Leaflet Ratings

On average, respondents who were given the educational leaflet (n = 246) rated it highly on a 1–6 scale, with higher scores reflecting more positive ratings across all 6 measures: useful (M ± SD = 5.1 ± 1.1), understandable (M± SD = 4.9 ± 1.3), accurate (M± SD = 5.0 ± 1.1), relevant (M = 4.1 ± 1.6), interesting (M± SD = 4.7 ± 1.4), and well-designed (M± SD = 4.9 ± 1.2).

## DISCUSSION

In this online randomized controlled survey experiment of 504 US adults aged ≥65 years, we found that a patient-centered educational leaflet significantly reduced reported desires to take antibiotics for ASB and decreased respondents' misperceptions of ASB as UTI. Conversely, respondents in the study who received an explicit recommendation from a surgeon to take antibiotics were less comfortable not taking antibiotics for ASB and more likely to misperceive their ASB as UTI. These findings offer important preliminary evidence that patient-centered educational materials can effectively prepare patients and their caregivers to participate in ASB-related treatment decisions. They also underscore the critical role of clinician recommendations aligning with the guidance presented in educational materials.

Respondents who received the leaflet had the highest levels of comfort with not taking antibiotics for ASB and the lowest rates of misperceiving ASB as UTI. Prior work has highlighted the importance of consulting with end users when developing health-related educational materials to ensure that they can respond to the needs and preferences of all who might interact with them [17]. The leaflet used in this study was designed with the input of patients and caregivers as well as health services researchers, psychologists, sociologists, and clinicians [30]. Our findings underscore the value of involving various end users in the design and development of patient-facing educational materials related to ASB and antibiotic use. The leaflet also improved knowledge of ASB, UTI, and antibiotics. Given the variable quality of publicly available information on UTI and ASB [29], this leaflet may serve as a valuable evidence-based online resource for broad use as well as a patient-facing tool in clinical settings. Moreover, our findings did not differ meaningfully by gender, suggesting that the leaflet may be broadly applicable. As with other cross-sectional surveys, we could not assess whether the observed effects would persist or strengthen with repeated exposure. Indeed, prior research shows that repeated messaging often amplifies impact [33] and even though many studies show changes in antibiotic preferences immediately after information exposure [34–37], some effects may only emerge or grow over a longer period than was covered in this study.

Despite these positive results, it is notable that a substantial proportion of respondents who received the educational leaflet still reported discomfort with not receiving antibiotics (44%), even though only 10% misperceived ASB as UTI based on the scenario. While there is clear scope for improving public knowledge about ASB, UTI, and antibiotics, these findings align with prior research showing that passive educational interventions alone often fail to effectively address antibiotic-related beliefs and intentions among a subset of people [34, 35]. It is possible that combining the leaflet with a surgeon's reassurance not to treat ASB could further reduce discomfort and misperceptions. However, our study did not assess the potential incremental benefit of the leaflet when paired with an appropriate recommendation, and this remains an important area for further study. It is also worth noting that for some respondents, their desires to take antibiotics following a positive urine test were not linked with the belief that they had a UTI. Future research should focus on identifying patient concerns that motivate them to want antibiotics for ASB that are not addressed by this educational leaflet and explore alternative strategies that may be more effective.

As expected, the surgeon's recommendation to take antibiotics had a negative impact on respondents' comfort with not taking antibiotics and increased misperceptions of ASB as UTI but did not influence general knowledge. Despite rising distrust in health care and science, health care professionals remain highly trusted by the public, and their recommendations continue to influence patients' behaviors and beliefs [38]. These findings underscore the importance of engaging clinicians to ensure that they believe in and are supportive of what educational materials say, so that they can in turn provide clear, transparent, and consistent guideline-aligned communication to patients.

One source of confusion regarding ASB treatment reported by older adults (≥65 years) and their caregivers is inconsistent communication from clinicians [17]. Our design allowed us to explore how conflicting information (ie, a surgeon recommending antibiotics while also providing a leaflet explaining that they are unnecessary) might affect respondents' antibiotic preferences, UTI beliefs, and general knowledge. However, we found no statistically significant interaction effects, suggesting that the contrasting effects (ie, the leaflet increasing comfort and the recommendation decreasing comfort) simply countered each other. While it is possible that the leaflet may have a protective effect against the surgeon's recommendation, our study was underpowered to detect this ordinal interaction. Moreover, any protective effect would likely be minimal and of limited clinical value, as we would expect that a clinician's recommendation during an actual consultation would likely have a much stronger influence on patients' beliefs and intentions than we observed in this study. These findings further point to the importance of clinician education and engagement as well as the practical challenges of implementing patient-focused educational interventions that conflict with a surgeons' recommendations. In real-world settings, it is unclear who would deliver the leaflet—the surgeon, a nurse, or another team member—and how that delivery would be perceived. Surgeons may be reluctant to distribute materials that contradict their own guidance or to allow others to intervene in their communication with patients. Thus, further work is needed to understand how to integrate the patient-facing educational leaflet into surgical workflows in ways that account for the social dynamics of the clinical team, minimize disruption to established communication patterns, and identify contingency plans for cases where the surgeon disagrees with the content or opts out of providing it.

We acknowledge several limitations of our study. First, the hypothetical nature of the scenarios presented to respondents does not fully replicate the emotional and physical reactions experienced by patients during clinical encounters and reflects a single clinical situation. While ASB can occur in varied clinical contexts and urine culture sampling before nonurologic procedures is not universally performed, we chose this preoperative scenario to provide a plausible context for evaluating the leaflet's impact without overburdening respondents. This approach also enabled us to assess the impact of the leaflet in a controlled experimental design and isolate its effects alongside the surgeon's recommendation. In addition, respondents in group 4 read a scenario where the surgeon recommended antibiotics but then handed them a leaflet with contrary information. While this represents an unlikely clinical situation [39], it allowed us to explore how respondents reacted to conflicting advice about the management of ASB [17]. Second, our findings rely on self-reported beliefs and intentions, which may not accurately reflect true thoughts or behaviors. However, the use of self-reported measures was necessary to directly capture the perspectives and preferences from a large sample of US adults within a short time frame for this experimental study. In addition, self-reported responses have been shown to correlate well with actual medical records and future health care behaviors [40, 41]. Furthermore, to enhance the reliability and validity of our data, we employed multiple verification strategies within the survey (eg, bot checkers and analysis of written responses) and included quotas to ensure adequate representation across key respondent characteristics. Third, we used an online survey platform to recruit respondents, which may have introduced selection and volunteer bias [42]. Specifically, our sample may reflect older adults who are more health-engaged or technologically adept, limiting generalizability to the broader adult population, especially older adults who may be less comfortable using online technology. Future studies with alternative recruitment strategies (eg, telephone or in-person surveys) would help capture a wider range of perspectives.

## CONCLUSIONS

We found that a patient-centered educational leaflet was effective in aligning the antibiotic treatment preferences of US adults age ≥65 years with clinical guidelines to avoid treatment of ASB with antibiotics and in improving knowledge about UTI, ASB, and antibiotics. These findings offer important preliminary evidence on the potential for patient-centered education to prepare patients and their caregivers to engage in ASB-related treatment decisions.

## Supplementary Material

ofaf690_Supplementary_Data

## Supplementary Data

Supplementary materials are available at *Open Forum Infectious Diseases* online. Consisting of data provided by the authors to benefit the reader, the posted materials are not copyedited and are the sole responsibility of the authors, so questions or comments should be addressed to the corresponding author.
