# Supplementary material for: Impact of an Educational Leaflet About Asymptomatic Bacteriuria and Urinary Tract Infection on Antibiotic Preferences Among US Adults ≥65 Years: An Online Randomized Controlled Survey Experiment
Source: Open Forum Infect Dis. 2025 Dec 15;12(12):ofaf690. doi: 10.1093/ofid/ofaf690 (PMC12703713; doi:10.1093/ofid/ofaf690)
Supplement: ofaf690_Supplementary_Data [file ofaf690_supplementary_data.docx]

**Supplement**

**Title:** Impact of an Educational Leaflet about Asymptomatic Bacteriuria And Urinary Tract Infection on Antibiotic Preferences Among US Adults ≥65 Years: An Online Randomized-Controlled Survey Experiment

**Authors:** Alistair Thorpe PhD^1^, Rachael A Lee MD, MSPH^2,3^, Julia E Szymczak PhD^4^, Madeline Farrell MSc, Karen Howard PharmD^5^, Brandi M. Muller MA^4^, Andrea T White PhD^6^, Angela Fagerlin PhD^1,7^, Valerie M Vaughn MD, MSc^6^

1. Department of Population Health Sciences, Spencer Fox Eccles School of Medicine at University of Utah, Salt Lake City, UT
2. Department of Medicine, Division of Infectious Diseases, UAB School of Medicine, AL
3. Department of Medicine, Division of Infectious Diseases, Birmingham VA Medical Center, Birmingham, AL
4. Department of Internal Medicine, Division of Epidemiology, Spencer Fox Eccles School of Medicine at University of Utah, Salt Lake City, UT
5. Department of Pharmacy, University of Utah Health, Salt Lake City, UT
6. Department of Internal Medicine, Spencer Fox Eccles School of Medicine at University of Utah, Salt Lake City, UT
7. Salt Lake City VA Informatics Decision-Enhancement and Analytic Sciences (IDEAS) Center for Innovation, Salt Lake City, UT

Corresponding author: Alistair Thorpe PhD; ([alistair.thorpe@hsc.utah.edu](mailto:alistair.thorpe@hsc.utah.edu)). Department of Population Health Sciences, Spencer Fox Eccles School of Medicine at University of Utah, 295 Chipeta Way, Room 1N476A, Salt Lake City UT 84108 | (801) 587-0742

**Supplement description**

This supplement contains the specific survey quotas, additional methodological details, GROVE reporting checklist, and the full list of survey items for the present study.

**Survey Quotas**

From March-April, 2024, respondents were recruited for this English-language survey by Dynata. Dynata are a commercial market research company with diverse pools of individuals who have agreed to be invited to take part in online survey studies (<https://www.dynata.com/>). This study was deemed exempt by the University of Utah IRB (IRB_00167676) with a waiver of informed consent granted and followed STROBE guidelines.

We used recruitment quotas for self-reported age, gender identity, racial/ethnic identity, and US census region to oversample underrepresented groups as follows:

| **Overall sample recruitment quotas** | | | | |
| --- | --- | --- | --- | --- |
| Age in years | |  | Gender identity | |
|  | 18-33 (22%) |  |  | Female (49%) |
|  | 34-49 (22%) |  |  | Male (49%) |
|  | 50-64 (22%) |  |  | Any other (1%) |
|  | ≥65 (34%) |  |  |  |
|  |  |  |  |  |
| Racial/Ethnic identity | |  |  | US Census region* |
|  | Non-Hispanic White (30%) |  |  | Northeast (17%) |
|  | Non-Hispanic Black (30%) |  |  | Midwest (21%) |
|  | Hispanic (30%) |  |  | South (39%) |
|  | Any other (10%) |  |  | West (24%) |

*US Census region quotas were chosen to reflect population estimates at the time of the survey according to <https://www.census.gov/topics/population.html>

**Additional methodological details**

*Missingness:* We did not perform any imputation methods because the missingness across study variables was infrequent and very low when present (<0.2% overall at most). Rates of missingness across study variables are reported in Table 1.

*Race and Ethnicity:* The race and ethnicity options pre-programmed into the survey were defined by the investigators but allowed all respondents the opportunity to self-describe if they wished. Race and ethnicity were included to allow us to describe the sample.

**Open text responses**

Open text responses can be viewed at: https://rpubs.com/AlistairThorpe/PRIME-UTI_OpenText

**Survey items**

**Study information:**

We invite you to participate in a research study about decisions you make regarding your health. In this study, you will be asked some questions about your personal opinions, previous decisions, and experiences regarding antibiotics, common infections, and COVID-19. We will also ask some questions about yourself. If you agree to participate, we would like you to answer the questions on the following screens.

It will take approximately 15 to 20 minutes to complete this survey. You are free to skip any questions that you prefer not to answer. Compensation for participation will be provided in accordance with your panel agreement.

Every effort will be made to protect your privacy and confidentiality. We will not collect your name or any identifying information about you. Your participation will be completely anonymous and it will not be possible to link you to your responses.

This study is not designed to benefit you directly. You have a choice about being in this study. You do not have to be in this study if you do not want to be.

The data we collect will be used for this study but may also be important for future research. Your data may be used for future research or distributed to other researchers for future study without additional consent if information that identifies you is removed from the data.

Taking part in this research study is completely voluntary. If you do not wish to participate in this study, simply click in the corner to close the web browser window.

You may have questions about your rights as someone in this study. If you have questions, you can call the University of Utah Institutional Review Board (the responsible Institutional Review Board) at 801-581-3655. Questions or concerns about this study or interest in the final results may be directed to Dr. Valerie Vaughn at valerie.vaughn@hsc.utah.edu. Thank you for taking part in this study.

**Demographics**:

What is your age?

Skip logic: if answer is <18 then skip to End of Survey.

How would you describe your gender identity?

- Female (1)
- Male (2)
- Transgender woman/Transwoman (3)
- Transgender man/Transman (4)
- Non-Binary/Third gender (5)
- Prefer not to say (6)
- Other please specify (7)

How would you describe your ethnic group or background? (Please select all that apply)

- American Indian or Alaskan Native (1)
- Asian or Asian American (2)
- Black or African American (3)
- Native Hawaiian or other Pacific Islander (4)
- White or European American (5)
- Other please specify (6)

Are you Hispanic or Latino/a or Latinx?

- No (1) | Yes (2)

What state do you live in?

↓ Alabama (1)…Wyoming (52)

Have you ever been prescribed antibiotics for a urinary tract infection?

- No (1) | Yes (2) | I don’t know (3)

Scenario: “Please read the text below carefully and imagine that the situation described is real.

Imagine you are planning to have a non-urgent surgery, perhaps a knee replacement or getting your tonsils out. You are asked to provide a urine sample during your pre-surgery office visit. The urine test comes back positive for bacteria. You are surprised as you do not have any symptoms of a urinary tract infection.

The surgeon comes in to see how you are feeling and talk with you about the surgery you have come in for. The surgeon explains the procedure, how long they expect it to take, and reviews your medical report and lab work.

*Based on the positive urine test result the surgeon recommends that you take a course of antibiotics.*

**Before leaving the room, the surgeon gives you a handout with some information about urinary tract infections and antibiotics [shown on the next page]**.”

- (Note: The text above represents the scenario presented to Group 3. Italics indicate the text that was removed for the control group and Group 2. Bold font indicates the text that was removed for the control group and group 1.

The three-page PDF educational leaflet about UTIs, ASB, and antibiotics. [only shown to groups 2 and 3]

How would you feel about NOT taking antibiotics in this situation?

- Very uncomfortable (1) | Uncomfortable (2) | Comfortable (3) | Very comfortable (4)
  *[if 1 or 2 selected] Could you tell us more about why you would feel that way? [Open text]*

Based on this scenario, do you think you have a urinary tract infection?

- No (1) | Yes (2) | Not sure (3)
- If someone has bacteria in their urine that means that they have a urinary tract infection
- Bacteria in the urine does not always need to be treated with antibiotics
- To confirm a bacterial urinary tract infection, you need to have both specific symptoms and a positive test for bacteria in the urine
- Symptoms like fever, confusion, feeling tired or dizzy, a change in color or smell of urine, or a fall could have many other causes and might not be a UTI.
- Disagree (1) | Agree (2) | Not sure (3)

How would you describe the information you saw? [shown only to those who saw the leaflet].

| Not at all useful-(1) | (2) | (3) | (4) | (5) | (6)-Very useful |
| --- | --- | --- | --- | --- | --- |
| Very difficult to understand-(1) | (2) | (3) | (4) | (5) | (6)-Very easy to understand |
| Very inaccurate-(1) | (2) | (3) | (4) | (5) | (6)-Very accurate |
| Not at all relevant to me-(1) | (2) | (3) | (4) | (5) | (6)-Very relevant to me |
| Not at all interesting to me-(1) | (2) | (3) | (4) | (5) | (6)-Very interesting to me |
| Very badly designed-(1) | (2) | (3) | (4) | (5) | (6)-Very well designed |

**Debrief:** Thank you for your participation! You are now finished with this survey. In this study, we are interested in understanding how to communicate about the risks of antibiotic resistance and people’s attitudes and understanding of antibiotics. We greatly appreciate all your responses! We are learning a lot about these topics, and we hope to share our findings broadly soon.

For accurate, up-to-date information please see the following CDC websites:

- Antibiotic resistance: <https://www.cdc.gov/drugresistance/index.html>
- Patient resources about antibiotics: <https://www.cdc.gov/antibiotic-use/materials-references/index.html>
- COVID-19: <https://www.cdc.gov/coronavirus/2019-ncov/index.html>

Thank you for participating in this survey.

**GROVE Checklist**

**Checklist form - Guideline for RepOrting Vignette Experiments (GROVE)***

Note: this document was originally published in the journal ‘Patient Education and Counseling’ under Creative Commons Attribution 4.0 International License (see <http://creativecommons.org/licenses/by/4.0/>). No changes were made to the original document, which was included as Appendix 1 to the following article: Hillen, M.A., Visser, N.C., Labrie, N.H.M. et al. Development of GROVE: a Guideline for RepOrting Vignette Experiments conducted in a healthcare context. Pat Educ Couns (2025). DOI: <https://doi.org/10.1016/j.pec.2025.108750>

| **Criterion** | **Description** | **Location in manuscript where item is reported** | **Details on methodological approach**** |
| --- | --- | --- | --- |
| **1. Rationale** | Provide a rationale for the use of an experimental vignette-based design, including an explanation why the study could not be conducted in a non-simulated setting. | - **Discussion: paragraph 6** | Our design allowed us to explore how providing an educational leaflet vs. surgeon recommending antibiotics might affect respondents’ antibiotic preferences, UTI beliefs, and general knowledge whilst also controlling for multiple factors (e.g., type of/reason for surgery, type of clinical setting, surgeon communication style, timing of intervention delivery) that might also influence responses, that we could not do to the same degree in a non-simulated setting. |
| **2. Vignette content** | Describe in detail how the vignette content was developed and refined, and explain any choices made. |  | *Report all relevant information below (sub-criteria 2.1-2.5)* |
| ***2.1. Clinical scenario*** | Describe and explain in detail how the healthcare scenario was developed and what it entailed. Include information about the sources used to inform vignette content, key characteristics of the portrayed characters, and the setting described in the vignette. | - **Procedure: paragraph 1** - **Figure 1** - **Supplement** | The scenario was designed by the study team to represent a situation in which, according to clinical guidelines from the Infectious Diseases Society of America, such a patient has ASB, not a UTI, and should not receive antibiotics. Respondents were shown a scenario describing themselves as an asymptomatic patient with a positive urine test during prescreening for a non-urgent, non-urologic surgery. They were then assigned to one of four conditions, which varied by educational leaflet provision and surgeons’ treatment recommendation for antibiotics. |
| ***2.2. Manipulation &   standardization*** | Describe what the experimental manipulations are (i.e., operationalization of the phenomenon under study), detailing which elements of the scenario were varied and how. Also report how other elements in the vignette were kept constant and provide information on vignette duration or length. | - **Procedure: paragraph 1** - **Figure 1** - **Supplement** | In a 2x2 between-group experimental design, we examined the effects of a three-page PDF educational leaflet about UTIs, ASB, and antibiotics (Not provided vs. Provided) alongside a surgeon’s treatment recommendation (None given vs. Recommended to take antibiotics). |
| ***2.3. Mode of delivery*** | Describe and explain the delivery modality and provide any information necessary for replication. Explain choices regarding narrative perspective and amount of detail described. Describe how participants were introduced to the vignette and in which setting data were collected. | - **Methods: paragraph 1** | The scenario was delivered to respondents in an online survey study and was viewed by respondents on their personal display system (e.g., monitor or laptop screen). |
| ***2.4. Expert   involvement*** | Explain who were involved in developing the vignettes, highlighting their particular expertise and contributions. | - **Procedure: paragraph 1** | The study team developed the scenario with targeted input from both clinicians and pharmacists |
| ***2.5. Pilot testing*** | Describe if, how, and when pilot testing was used in the vignette development process. Explain whether and how this affected the vignette content and format. | **N/A** | No pilot testing was conducted with the vignette |
| **3. Outcomes &   participant   instructions** | Explain the selected study outcome(s) for the vignette study, particularly how these outcome(s) relate to real-world outcomes of interest. | - **Measures** | The primary outcome was respondents’ comfort with not taking antibiotics for ASB. Secondary outcomes were reported misperceptions of ASB as UTI and knowledge. These measures relate directly to patient preferences, desires, beliefs, and intentions which are known to influence their health behaviors. |
| **4. Vignette validity &   realism** | Report how manipulation success of the independent variable(s) of interest was evaluated (i.e., manipulation check). Also describe if and how realism and aspects of participant engagement with the scenario were assessed. | - **Sample characteristics: paragraph 1** | No specific manipulation checks were included. Respondent engagement with the scenario was assessed via responses to open-text questions. |
| **5. Participants** | Provide a rationale for the choice of study participants (e.g., analogue patients), both in relation to the target population and to the characters portrayed in the vignettes. | - **Introduction: paragraph 2** | Our sample of US adults ≥65 years was chosen because this patient population is likely to have ASB and to be unnecessarily treated. Thus the situation described in this vignette is clinically relevant to this population. |
| **6. Accessibility** | Include information on the availability of the final vignettes and pilot data for research, teaching, or commercial purposes. Detail any restrictions to access and (re)use of the vignettes and data. | - **Procedure: paragraph 1** - **Supplement** | The complete final vignettes are provided in the supplementary materials. Access and reuse of the vignettes and associated data are permitted in accordance with the terms of the publishing license. |

*The order of reporting these criteria is intended to be flexible. Information can be combined or reorganized and information may be placed in any manuscript section, figure, table and or supplementary material, depending on the study content and journal requirements.

**Authors may report additional methodological details in this column beyond the information included in their main manuscript.
